# CLOSE-UP – a favourable protocol for limb-sparing surgery of diabetic foot osteomyelitis

**DOI:** 10.5194/jbji-10-199-2025

**Published:** 2025-06-16

**Authors:** Anton Alexander Nolte Peterlin, Louise Kruse Jensen, Emil Gleipner-Andersen, Hans Gottlieb

**Affiliations:** 1 Experimental Pathology, Department of Veterinary and Animal Sciences, University of Copenhagen, 1870 Frederiksberg, Denmark; 2 Department of Orthopaedic Surgery, Herlev Hospital, 2730 Herlev, Denmark

## Abstract

**Introduction**: Diabetic foot osteomyelitis (DFO) is a severe complication of diabetic foot ulcers, leading to high morbidity, mortality, and major limb amputation risk. While limb-sparing surgery is well established, optimal wound closure and intraosseous antibiotic strategies remain under-explored and under-reported. This study evaluates a single-stage limb-sparing surgical approach incorporating primary closure and local intraosseous antibiotic therapy. **Methods**: This retrospective study included 97 DFO patients (2017–2024) treated using the CLOSE-UP (Conservative surgery, Local antibiotics, Oral versus intravenous antibiotics – OVIVA, Samples, Effective limb preservation, and closUre Primary) protocol, developed to standardize DFO surgery. The one-stage procedure involved bone sampling, local debridement or minor amputation (distal to the tarsometatarsal joint), antibiotic-loaded calcium sulfate–hydroxyapatite biocomposite application, and primary wound closure. Postoperatively, patients followed the OVIVA antimicrobial protocol: 1 week of intravenous (IV) therapy and 5 weeks of oral (empiric penicillin–cloxacillin) therapy. The primary outcome was treatment failure within 1 year, with a minimum follow-up of 12 months. **Results**: Clinical failure occurred in 13 patients (13.4 %), with only 4 patients (4.1 %) requiring major amputation. Peripheral arterial disease was present in 24 patients (24.7 %) and was the only variable significantly associated with clinical failure (odds ratios: 10.21; P<0.01 ). The 1-year and 3-year mortality rates were 14.4 % and 35.9 %, respectively. **Conclusions**: The CLOSE-UP protocol demonstrated favourable outcomes. Given the high risk of mortality and limb loss in DFO, this structured approach has the potential to improve mobility, shorten rehabilitation, lower costs, and enhance quality of life. Further research, particularly randomized controlled trials, should focus on optimizing wound closure to improve long-term limb preservation and survival.

## 1 Introduction

Diabetic-related osteomyelitis of the foot (DFO) is a common complication in diabetic patients, with a reported prevalence of 15 % (Lázaro Martínez et al., 2019). The pathogenesis is well documented, typically arising from diabetic foot ulcers and spreading contiguously to the bone (Aragón-Sánchez and Lipsky, 2018; Lipsky and Uçkay, 2021; Uçkay et al., 2015). If left untreated, ulcers can progress to DFO, which is the leading cause of non-traumatic major lower-limb amputations and is associated with significant morbidity and mortality (Tay et al., 2019). According to the same study, early detection and appropriate treatment may prevent up to 85 % of these amputations.

DFO is managed through a combination of wound care, antibiotic therapy, and surgery. However, surgical approaches remain heterogeneous. Increasing evidence supports prioritizing conservative, limb-sparing techniques to reduce the need for major amputations (above the ankle) while also preserving function and improving patient outcomes (Aragón-Sánchez and Lipsky, 2018; Lázaro Martínez et al., 2019; Senneville et al., 2024). These strategies often involve debridement of infected bone and nonviable soft tissue, with or without partial amputation distal to the ankle joint (minor amputation) (Aragón-Sánchez et al., 2023; Blanchette et al., 2022; Nguyen et al., 2022; Schöni et al., 2023). When employed, this approach has shown clinical failure rates ranging from 0 % to 36 % (Aragón-Sánchez et al., 2008, 2023; Blanchette et al., 2022; Faglia et al., 2012; Nguyen et al., 2022; Schöni et al., 2023). A critical factor influencing the success of these strategies is wound closure, which can be achieved through primary, delayed-primary, or secondary intention, usually depending on the extent of soft-tissue infection (Fisher et al., 2010). Despite its importance, wound closure is often overlooked or inconsistently reported in DFO study methodologies (Aragón-Sánchez et al., 2008, 2023; Blanchette et al., 2022; Faglia et al., 2012; García-Morales et al., 2012; Kowalski et al., 2011; Schöni et al., 2023; Winkler et al., 2022). Primary closure has been associated with faster healing, reduced exudation, and similar reinfection rates compared to healing by secondary intention (García-Morales et al., 2012).

In addition to debridement and wound closure techniques, local antimicrobial therapy can play a crucial role in optimizing surgical outcomes, particularly in cases with impaired systemic antibiotic delivery, such as in patients with peripheral arterial disease (PAD) (Raymakers et al., 2001). Local intraosseous antibiotics offer a promising strategy for managing dead space after debridement, delivering high concentrations of drugs directly to the affected area while bypassing compromised circulation (Faglia et al., 2012; Venkateswaran et al., 2024).

Building on these principles, this study aims to evaluate the effectiveness of a single-stage limb-sparing surgical approach for DFO, incorporating primary wound closure and local intraosseous antibiotic therapy in 97 patients.

## 2 Methods

This retrospective cohort study included adult patients (aged ≥ 18 years) with DFO who underwent limb-sparing surgical treatment between April 2017 and August 2024. The diagnosis of osteomyelitis was established based on a positive probe-to-bone test and findings on plain radiographs. When necessary, magnetic resonance imaging was performed. This approach follows the criteria outlined in the Infectious Diseases Society of America guidelines (Lavery et al., 2007; Tay et al., 2019). Treatment was managed by a specialized multidisciplinary team (MDT) from the Department of Infection Surgery at Herlev Hospital, Herlev, Denmark. Surgery was performed by orthopaedic and plastic surgeons, antibiotic stewardship was led by clinical microbiologists and infectious disease specialists, and follow-up was provided in dedicated outpatient clinics by orthopaedic surgeons in collaboration with plastic surgeons, specifically for patients with bone and joint infections (BJIs). The minimum follow-up period was 12 months. To ensure independent observations, only one episode of DFO per patient was included in the study. For patients with multiple episodes, only the first occurrence was considered.

If patients had palpable dorsal pulses, no further vascular assessment was performed. In the absence of palpable pulses, additional testing was conducted. PAD was defined as a toe pressure of < 50 mmHg or an ankle–brachial index (ABI) < 0.9. All patients with PAD were evaluated by a vascular surgeon and, if possible, optimized preoperatively. Patients unable to be revascularized and/or with a toe pressure < 30 mmHg and an ABI of < 0.4 underwent a primary above-ankle amputation and were excluded from the study. Infection severity was assessed clinically as part of routine care; formal scoring systems (e.g. IWGDF/IDSA or WIfI) were not applied. Some patients also received antibiotics prior to surgery, although this was not systematically recorded. Patient demographics included sex, body mass index, cardiovascular disease (including myocardial infarction, angina pectoris, and congestive heart failure), PAD, the American Society of Anesthesiologists score, and a history of tobacco and alcohol use.

### 2.1 Surgical and patient management

**Figure 1:**
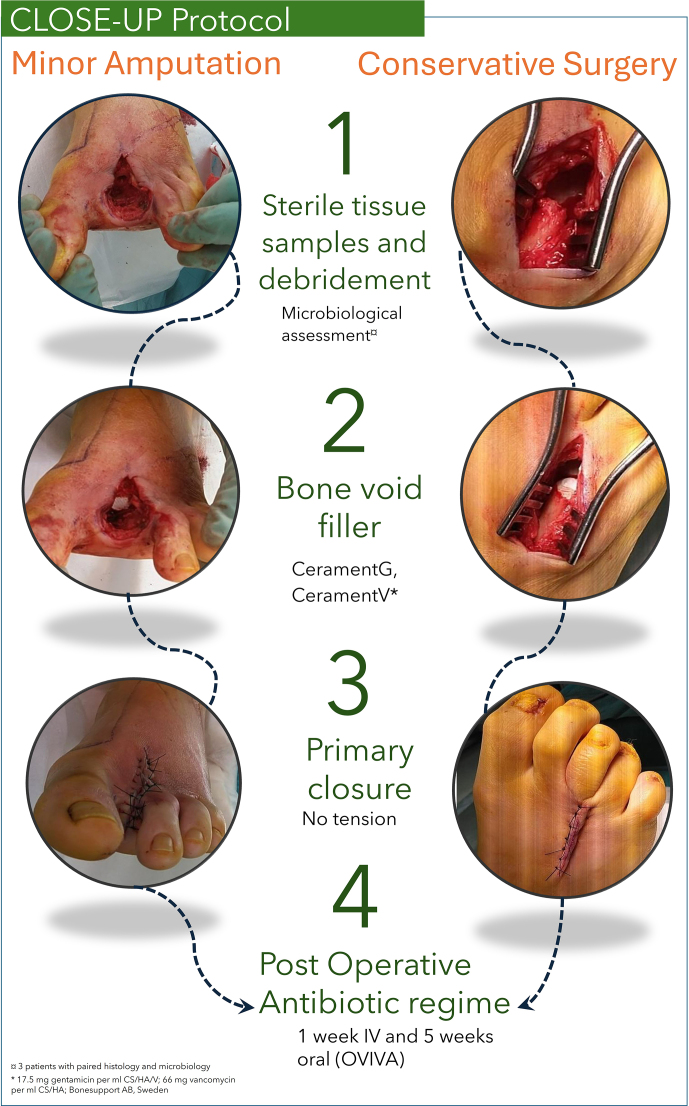
The CLOSE-UP surgical algorithm for DFO management.

The CLOSE-UP (Conservative surgery, Local antibiotics, Oral versus intravenous antibiotics – OVIVA, Samples, Effective limb preservation, closUre Primary) protocol was developed to standardize DFO surgery. This one-stage, limb-sparing approach involved either (1) debridement of infected bone and nonviable soft tissue without amputation or (2) minor amputation distal to the tarsometatarsal joint (Aragón-Sánchez et al., 2023). Five deep-tissue specimens were collected per patient for microbiological culture, each using a separate sterile instrument (Dudareva et al., 2021a). Samples were taken either proximally to the resected bone (e.g. from the proximal margin of the metatarsal head in a first-ray amputation) or directly from the infected bone. Residual bone voids were filled under a short-duration tourniquet with an antibiotic-eluting calcium sulfate–hydroxyapatite graft substitute (CERAMENT G: 17.5 mg gentamicin mL^−1^; CERAMENT V: 66 mg vancomycin mL^−1^; BONESUPPORT AB, Sweden). All wounds were closed primarily without skin tension (Fig. 1). CERAMENT G was the default bone void filler; however, if the identified pathogen was resistant to gentamicin, CERAMENT V was used instead. Postoperatively, patients received 6 weeks of systemic antibiotics – 1 week of intravenous (IV) therapy followed by 5 weeks of oral therapy – aligned with the OVIVA trial findings (Li et al., 2019). This regimen represents the standard postoperative treatment for all BJIs at Herlev Hospital. Empirical therapy included IV penicillin G (1200 mg) and dicloxacillin (1000 mg) every 6 h, followed by oral penicillin V (1200 mg) and dicloxacillin (1000 mg). Antibiotic regimens were adjusted based on culture sensitivity. Post-discharge, patients were monitored in an outpatient clinic by specialized nurses and doctors.

### 2.2 Outcomes

The primary outcome was clinical failure, defined as the need for revision surgery, including further debridement for a non-healing wound, a persistent sinus tract at the surgical site, or any proximal amputation within 12 months of the initial intervention (Gill et al., 2022; Lebowitz et al., 2017; Schöni et al., 2023). Secondary outcomes included mortality and the microbiological profile.

### 2.3 Statistical analyses

Baseline characteristics were summarized using the median (Q1–Q3) for continuous variables and n (%) for categorical variables. Only patients with clinical failure or a minimum of 1 year of follow-up were included in these analyses. Univariate logistical regression was performed to assess the risk factors for clinical failure, with results reported as the odds ratio (OR) with the 95 % confidence interval (CI). Variables with P<0.20 in univariate analysis were included in the multivariate logistic regression model to minimize the risk of prematurely excluding potential confounders. The association between microbiology findings and clinical failure was assessed using Fisher's exact test. Mortality was assessed using multivariable Cox regression and Kaplan–Meier estimates. All analyses were conducted in R version 4.2.3 (R Core Team, 2024). P values < 0.05 were considered statistically significant.

## 3 Results

### 3.1 Characteristics of the study population

A total of 97 patients were included in the study (Table 1), with 24.7 % diagnosed with PAD. Nine patients (9.3 %) underwent surgical debridement without amputation, while the majority (90.7 %) required minor amputation. The distal or proximal phalanx of the great toe was the most affected site. The most frequent minor amputation was first-ray amputation, performed in 40 cases (45.5 %), followed by second-ray amputation in 24 cases (27.3 %). Amputation rates for the remaining toes were similar (9.1 %). Conservative surgical approaches were most used for the distal phalanx of the hallux (44.4 %) and the base of the fifth metatarsal (33.3 %). As a local bone void filler, CERAMENT G was used in 96 patients, while CERAMENT V was administered to a single patient. Of the 41 patients who completed the full course of empirical antibiotic therapy, only 3 experienced treatment failure. Among the remaining patients, systemic antibiotic treatment was also completed; however, 13 never initiated empirical therapy due to known allergies, 40 had their regimens adjusted based on microbiological susceptibility, and 3 had changes made due to other illnesses (such as pneumonia). In this group, 10 patients experienced treatment failure.

**Table 1:** Baseline and clinical characteristics of the study population, including clinical failure outcomes ( N=97 ).

	Value
Age, years	68 (59–76)
BMI, kg m^−2^	28 (24–31)
Male sex	77 (79.4)
Follow-up, years	2.2 (1.5–3.5)
ASA score	
2	16 (16.5)
3	75 (77.3)
4	6 (6.2)
Cardiovascular disease	76 (78.4)
Peripheral arterial disease	24 (24.7)
Smoker	63 (64.9)
Conservative surgery	9 (9.3)
Clinical failure	13 (13.4)
Reoccurrence	7 (7.2)
Revision	2 (2.1)
Major amputation	4 (4.1)
Time to failure, days	35 (15–56)
Completed empirical antibiotics	41 (42.3)

Data are presented as n (%) or median (Q1–Q3). Abbreviations used in the table are as follows: BMI – body mass index; ASA – American Society of Anesthesiologists.

### 3.2 Clinical failure and risk factors

Clinical failure occurred in 13 patients (13.4 %), with only 4 (4.1 %) requiring major amputation. PAD was the only variable significantly associated with clinical failure (OR: 10.2; 95 % CI: 2.7–38.3; P<0.01 ; Table 2). No other variables, including completion of empirical treatment or microbiology grouping, were associated with clinical failure ( P=0.12 and P=0.16 , respectively; Table 2).

**Table 2:** Risk factors for clinical failure.

Variable	OR (95 % CI)	P value
Male sex	3.2 (0.38–26.3)	0.29
Tobacco	1.4 (0.40–5.1)	0.58
Peripheral arterial disease	10.2 (2.7–38.3)	< **0.01**
ASA		
2	0.75 (0.15–3.8)	0.73
3	0.95 (0.23–3.9)	0.95
4	2.9 (0.24–34.7)	0.39
Microbiology grouping		
Gram-positive	0.32 (0.07–1.6)	0.16
Polymicrobial	1.4 (0.39–5.3)	0.58
Gram-negative	1.1 (0.22–5.9)	0.89
Culture-negative	1.7 (0.51–6.0)	0.38
Completed empirical treatment	0.34 (0.09–1.3)	0.12

Abbreviations used in the table are as follows: OR – odds ratio; CI – confidence interval; ASA – American Society of Anesthesiologists. Boldface indicates statistical significance ( P<0.05 ).

### 3.3 Microbiology

Overall, 25.8 % ( n=25 ) of cases were culture-negative, of which 22 (88 %) were from proximal resected bone margins; 26.8 % had polymicrobial infections; 13.4 % had Gram-negative bacteria; and 34.0 % had Gram-positive bacteria (Table 3). The microbiological distribution did not differ significantly between clinical success and failure groups (Table 4). The median number of positive biopsies was 4 (Q1–Q3: 2–7) in the clinical failure group and 2.5 (Q1–Q3: 1–5) in the non-failure group, although this difference was not statistically significant ( P=0.95 ). The only resistant organism found in this study was a carbapenemase-producing *Enterobacter cloacae* (Table 3).

**Table 3:** Microbiological profile of isolates from diabetic foot osteomyelitis cases, 153 isolates.

Group/family/species	Group/family frequency	Species frequency
Gram-positive aerobic bacteria	115 (75.2)	
Staphylococcaceae family	72 (47.1)	
*Staphylococcus aureus*		24 (15.7)
*Staphylococcus epidermidis*		22 (14.4)
*Staphylococcus lugdunensis*		2 (1.3)
Other CoNS^1^: *Staphylococcus simulans,*		24 (15.7)
*Streptococcus anginosus, Streptococcus haemolyticus,*		
*Staphylococcus capitis, S. hominis, S. warneri,*		
*S. pettenkoferi*, and *S. pateuri*		
The α - and β -hemolytic Streptococcaceae family	12 (7.8)	
Group-B streptococci		6 (3.9)
α -hemolytic, *Streptococcus sanguinis*, and *S. mitis*		5 (3.3)
Other: *Streptococcus canis,*		1 (0.7)
The γ -hemolytic Streptococcaceae family	12 (7.8)	
*Enterococcus faecalis*		11 (7.2)
*Enterococcus avium*		1 (0.7)
*Corynebacterium* sp.	11 (7.2)	
Additional Gram-positive species	8 (5.2)	
Gram-negative aerobic bacteria	37 (24.2)	
Enterobacteriaceae family	23 (15.0)	
*Escherichia coli*		6 (3.9)
*Citrobacter koseri*		1 (0.7)
*Klebsiella aerogenes* and *Enterobacter cloacae* ^2^		10 (6.5)
*Klebsiella oxytoca* and *K. pneumoniae*		6 (3.9)
Other families	11 (7.2)	
*Pseudomonas aeruginosa*		1 (0.7)
*Haemophilus parainfluenzae*		1 (0.7)
Morganellaceae (*Proteus* spp. and *Morganella morganii*)		6 (3.9)
*Neisseria subflava*		1 (0.7)
Actinobacter species		2 (1.3)
Anaerobic bacteria	3 (2.0)	
*Prevotella* spp.		3 (2.0)
Fungi	1 (0.7)	
*Candida albicans*		1 (0.7)

^1^ CoNS denotes coagulase-negative staphylococci. ^2^ One carbapenemase-producing *Enterobacter cloacae*. Data are shown as n (%).

**Table 4:** Microbiological findings in clinical success and failure groups.

	Clinical success ( n=84 )	Clinical failure ( n=13 )	P value
*Staphylococcus aureus*	11 (13.1)	1 (7.7)	1.00
*Staphylococcus epidermidis*	8 (9.5)	1 (7.7)	1.00
Other CoNS^*^	7 (8.3)	0 (0.0)	0.59
Other Gram-positive	5 (4.8)	0 (0.0)	1.00
Polymicrobial	22 (26.2)	4 (30.8)	0.74
Gram-negative	11 (13.1)	2 (15.4)	0.68
Culture-negative	20 (23.8)	5 (38.5)	0.33

^*^ CoNS denotes coagulase-negative staphylococci. Data are shown as n (%).

### 3.4 Survival analysis and mortality

**Figure 2:**
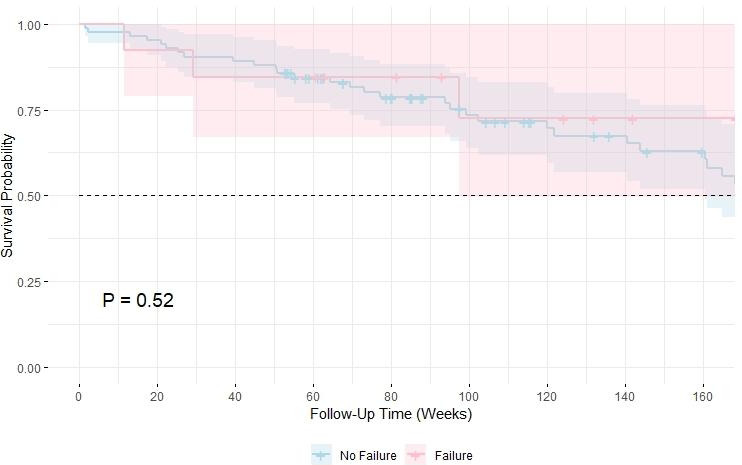
The 3-year survival probability comparing no failure (blue) and clinical failure (red), with the dashed line indicating the 50 % threshold. Survival estimates account for censoring and reflect cumulative mortality over time.

A Kaplan–Meier survival analysis comparing clinical failure and non-failure groups showed no difference in survival probabilities ( P=0.52 ) (Fig. 2a). The 1-year and 3-year mortality rates were 14.4 % (14) and 35.9 % (36), respectively. The 5-year mortality estimate was not reported due to the limited number of patients remaining in follow-up at this time point ( n=6 ), making the survival estimate unreliable (Fig. 2b). In a sub-group analysis, the PAD group had lower survival, but the difference was not statistically significant ( P=0.13 ). Mortality was higher in PAD patients (12 of 24; 50.0 %) compared to non-PAD patients (22 of 73; 30.1 %).

## 4 Discussion

This study highlights the effectiveness of the CLOSE-UP protocol – a novel standardized approach to the surgical management of DFO. With a failure rate of 13.4 %, these results fall at the lower end of the reported ranges for limb-sparring DFO surgery (0 %–36 %) (Aragón-Sánchez et al., 2008, 2023; Blanchette et al., 2022; Faglia et al., 2012; Nguyen et al., 2022; Schöni et al., 2023). Given the significant 3-year mortality rate (36 %) in this highly comorbid population (Fig. 2b), further evaluation of surgical strategies, including wound closure techniques, is warranted to optimize long-term limb preservation (Mponponsuo et al., 2021).

Current literature on DFO management often lacks detailed documentation of wound closure techniques. One study of 34 patients reported that primary closure led to significantly faster healing (9.9 ± 8.4 weeks) compared to healing by secondary intention (19.1 ± 16.9 weeks) (García-Morales et al., 2012). However, many studies emphasize limb-sparing surgery but fail to report wound closure methods consistently or do so with significant variability (Aragón-Sánchez et al., 2008, 2023; Baek et al., 2024; Faglia et al., 2012; Schöni et al., 2023). Additionally, multiple studies found no significant difference in failure rates between conservative surgery and minor amputations as primary endpoints, yet wound closure strategies varied widely. Schöni et al. (2023) and Aragón-Sánchez et al. (2008) allowed all wounds to heal by secondary intention, whereas Faglia et al. (2012) differentiated closure methods based on surgical type – using primary closure for conservative surgery and secondary intention for minor amputations – reporting similar failure rates of 13.6 % and 10.4 %, respectively.

Comparisons are further complicated by differences in study methodology. Baek et al. (2024) pooled major and minor amputations, with the latter showing a high failure rate of 41 % (21/51) for primary closure. Furthermore, Kowalski et al. (2011) reported that patients with positive resection margins, whose wounds were left to heal by secondary intention, had a higher risk of treatment failure. To address this gap, the CLOSE-UP protocol was implemented, standardizing the surgical approach to DFO, which resulted in low failure rates and favourable clinical outcomes, supporting the viability of primary closure in selected patients.

The data also highlight the clinical relevance of standardized microbiological sampling, as nearly 75 % (71/97) of cases had positive cultures (Dudareva et al., 2021b; Senneville et al., 2024; Travers et al., 2021). Despite the high percentage of positive bone margins, only PAD had a significant negative impact on clinical outcomes, consistent with existing literature (Kowalski et al., 2011; Senneville et al., 2024). These findings further validate the OVIVA protocol as the foundation for post-surgical antibiotic management and align with IWGDF (International Working Group on the Diabetic Foot) guidelines, which emphasize individualized, evidence-based antibiotic therapy (Li et al., 2019; Senneville et al., 2024).

Given that PAD and critical limb ischemia impair systemic antibiotic delivery, localized antimicrobial strategies could play a crucial role in infection management (Marson et al., 2018; Venkateswaran et al., 2024). The ongoing SOLARIO (Short or Long Antibiotic Regimes in Orthopaedics) trial introduces the possibility that systemic antibiotic therapy for bone and joint infections may be reduced to as little as 1 week when combined with local antibiotic treatment (Dudareva et al., 2019). This further emphasizes the importance of local antimicrobial strategies and the implementation of future programmes and studies, particularly in the management of diabetes-related foot osteomyelitis.

Similarly, a systematic review on local antibiotic use in diabetic foot infections also demonstrated a reduced treatment duration and reduced reoperation rates (Marson et al., 2018). The largest case series, by Gualand (2011), reported an 86 % healing rate without additional antibiotics; however, wounds healed by secondary intention, and all patients had an intact vascular supply. Another comparative study of 53 patients using local CERAMENT G or CERAMENT V combined with systemic antibiotics found no differences in clinical failure rates between groups, although once again none of the wounds were closed primarily (Venkateswaran et al., 2024). However, both the systematic review and the IWGDF guidelines conclude that, while local antibiotic therapy may be considered in cases where systemic delivery is compromised (such as in PAD), there is currently insufficient high-quality evidence to support the routine use of local antibiotic therapy over systemic antibiotics (Marson et al., 2018; Senneville et al., 2024).

The present study has several limitations, including its retrospective design, single-centre setting, no use of histopathology, and lack of standardized infection severity grading (e.g. IWGDF/IDSA or WIfI classification) and registration of pre-antibiotic use. Wound healing time was not consistently documented, and no comparative group undergoing secondary intention healing was included. Furthermore, patients with critical limb ischemia who could not be optimized before surgery underwent major amputation, introducing selection bias. However, all other patients with surgery demanding DFO who came to the hospital in the reported study period were included, keeping the selection and sample size bias at a minimum. The mentioned limitations may reduce comparability with other studies, but they also highlight the need for future research – particularly a randomized controlled trial evaluating wound closure strategies and their clinical impact.

## 5 Conclusions

This study demonstrates that DFO can be effectively managed with a one-stage, limb-sparing approach through the CLOSE-UP protocol, utilizing primary closure and yielding favourable outcomes. PAD was a significant risk factor for failure. Given the high mortality and major amputation risk in DFO patients, this approach can reduce limb loss; preserve mobility; and, assumably, shorten rehabilitation, lower healthcare costs, and ultimately improve quality of life. Further research, particularly randomized controlled trials, should focus on optimizing wound closure to enhance long-term limb preservation and survival.

## Data Availability

The data presented in this study are available upon request from the corresponding author. The data are not publicly available due to privacy concerns regarding protected health information. Statistical analyses were performed using R, version 4.2.3, and the code is available upon request (https://www.R-project.org/, R Core Team, 2024).
